# Interaction Between 5-HTTLPR Genotype and Cognitive Stress Vulnerability on Sleep Quality: Effects of Sub-Chronic Tryptophan Administration

**DOI:** 10.1093/ijnp/pyu057

**Published:** 2015-01-31

**Authors:** Jens H. van Dalfsen, C. Rob Markus

**Affiliations:** Department of Neuropsychology and Psychopharmacology, Faculty of Psychology and Neuroscience, Maastricht University.

**Keywords:** 5-HTTLPR, depression, sleep, stress, tryptophan

## Abstract

**Background:**

Abundant evidence suggests that allelic variation in the serotonin transporter-linked polymorphic region (5-HTTLPR) influences susceptibility to stress and its affective consequences due to brain serotonergic vulnerability. Based on recent assumptions, the present study examined whether the 5-HTTLPR genotype may also interact with a vulnerability to chronic stress experience (conceptualized by trait neuroticism) in order to influence sleep quality and, additionally, whether this is influenced by brain serotonergic manipulations.

**Methods:**

In a well-balanced experimental design, homozygous S-allele (n = 57) and L-allele (n = 54) genotypes with high and low chronic stress vulnerability (neuroticism) were first assessed for general past sleep quality during a month before onset of the experiment. Then subjects were assessed for sleep quality following 7 days of tryptophan (3.0g/day) or placebo intake.

**Results:**

Although high neuroticism was significantly related to a higher frequency of stressful life events and daily hassles, it did not interact with the 5-HTTLPR genotype on general past sleep quality. However, as expected, a 7 day period of tryptophan administration was exclusively associated with better sleep quality scores in the S’/S’ genotype with high trait neuroticism.

**Conclusions:**

Current findings suggest that 5-HTTLPR does not directly interact with stress vulnerability in order to influence sleep quality. Instead, based on current and previous findings, it is suggested that the S’/S’ 5-HTTLPR genotype promotes the risk for stress-related sleep disturbances because of an increased susceptibility to the depressogenic consequences of stress. Accordingly, by way of reducing depressive symptomatology, tryptophan augmentation may particularly improve sleep quality in stress-vulnerable individuals carrying the 5-HTTLPR S-allele.

## Introduction

Sleep disturbances are highly prevalent. In Western Europe around 30% percent of the general population report sleep problems (Leger et al., 2008). Such sleep difficulties have a high clinical significance, as they play an important role in both physical and psychological well-being (Lund et al., 2010). For example, sleep complaints have been associated with an increased risk of hypertension (Guo et al., 2013), cardiovascular disease (Schwartz et al., 1999), and diabetes (Beihl et al., 2009), as well as with various psychiatric disorders (Krystal, 2006).

There is large consensus that stress plays an important role in both the aetiology and persistence of sleep disturbances. For instance, sleep quality has been found to vary as a function of daily stressful life events (Vahtera et al., 2007; Mezick et al., 2009; Barclay et al., 2011a; Åkerstedt et al., 2012) and, hence, personality traits associated with stress vulnerability, like neuroticism, appear to be important predictors for sleep disturbances (Calkins et al., 2012).

In addition to studies revealing relationships between stress experiences and sleep difficulties, there are also studies investigating direct interrelationships between sleep quality and activation of the hypothalamic-pituitary-adrenal (HPA) axis: the major neuroendocrine system involved in stress responses, and, hence, stress adaptation (Markus, 2008). In general, increased HPA activation is found to impair sleep; most likely by increasing cortical arousal, resulting in lighter sleep and more nocturnal awakenings (Rodenbeck et al., 2002; Steiger, 2002; Buckley and Schatzberg, 2005). These findings suggest that the negative effects of stress experience on sleep quality may, at least in part, be mediated by HPA alterations.

Taking into account the importance of stress in the onset and course of sleep disturbances, there might be a particular moderating role for the brain’s serotonergic system. Brain serotonin (5-hydroxitryptamine [5-HT]) acts as a neurobiological mechanism for stress adaptation, as a stress-induced 5-HT increase is found to be involved in the negative feedback control of the HPA function and, hence, in regaining psychological balance after stress-induced alterations in HPA activation (Firk and Markus, 2007; Markus, 2008). Therefore, it is not surprising that reduced brain 5-HT functioning is frequently implicated as a risk factor for stress-related affective disorders such as depression, as further supported by the 5-HT mechanism of action of most antidepressant drugs (van Praag, 2004; van Praag et al., 2004; Markus, 2008).

Given the association between stress and sleep complaints and, hence, the importance of 5-HT in stress resilience, it is reasonable to assume that individual differences in 5-HT functioning may accordingly moderate differences in vulnerability for stress-related sleep complaints. Research suggests that 5-HT activation and/or functioning is, at least in part, genetically influenced, involving the 5-HT transporter-linked polymorphic region (5-HTTLPR). This polymorphism includes an allelic variation, of which the short (S) allele is associated with lower transcriptional efficiency compared to the long (L) allele. As a consequence, the S-allele is accompanied by a reduced number of 5-HT transporters, and, hence, lower 5-HT functioning (Heils et al., 1996; Lesch et al., 1996). In compliance with the role of 5-HT in stress adaptation, S-allele carriers are, indeed, generally found to respond with greater behavioral and HPA stress responses compared to L-allele carriers (Gotlib et al., 2008; Mueller et al., 2010; Way and Taylor, 2010; Cerit et al., 2013; Miller et al., 2013) and, hence, have an increased risk for depressive symptoms in response to stress (Caspi et al., 2003; Karg et al., 2011; Miller et al., 2013).

Based on previously-described relationships between stress and sleep, and between 5-HTTLPR and stress vulnerability, an intriguing possibility is that genetically 5-HT–vulnerable subjects carrying the S-allele are more prone to experience sleep difficulties as a function of high-stress experiences. To date, only a few studies have investigated this interaction between 5-HTTLPR and stress on sleep quality, revealing either indirect evidence (Brummett et al., 2007) or no evidence at all (Barclay et al., 2011b). In these studies, however, stress experience was either not directly measured (Brummett et al., 2007) or just conceptualized by the frequency of self-reported past-year life events in association with past-month sleep disturbance (Barclay et al., 2011b). Of course, the past life–event checklist approach does not account for individual differences in the experienced emotional impact an event might have.

In order to elucidate whether the frequent experience of negative stress specifically moderates the relationship between 5-HTTLPR and sleep quality, a more liable measure should be incorporated. Hence, according to the cognitive, vulnerability-transactional stress model of stress and depression (Lazarus and Folkman, 1984), life events particularly cause mental stress when they are actually appraised as personally relevant (primary appraisal) but unmanageable due to perceived insufficient coping abilities (secondary appraisal; Brown et al., 1987; Gunthert et al., 1999). Among the personality traits related to such a stress vulnerability, trait neuroticism has been the most recognized (van Praag, 2004; Shoji et al., 2010). Specifically, individuals with high trait neuroticism are more likely to frequently experience stress with negative emotional consequences (Watson and Clark, 1984; Luteijn and Bouman, 1988; Gallagher, 1990; van Praag, 2004), show low expectations for self efficacy, possess less-adaptive coping strategies for stress events (Gunthert et al., 1999; Penley et al., 2002; Shoji et al., 2010), and are more vulnerable for development of major depression (Roberts and Kendler, 1999; van Praag, 2004). Thus, although trait neuroticism is, as a personality trait, by definition not synonymous with stress experience, as a consequence it is thought of as a most adequate measure for the vulnerability to frequent, chronic experiences of stress and its negative emotional impact (Markus, 2013).

 From both a clinical and scientific perspective, it might be additionally useful to examine the influence of 5-HT brain augmentation on stress-related sleep difficulties. Moreover, as S-allele carriers are thought to be 5-HT vulnerable and, hence, have increased stress-vulnerability due to reduced 5-HT functioning (Markus et al., 2012), it might be expected that 5-HT brain augmentation is especially beneficial in reducing stress-induced sleep disturbances within this genotype. Research indicates that synthesis and release of brain 5-HT can be enhanced by increasing the availability of its precursor, tryptophan (TRP; Markus et al., 2000). This increase in brain 5-HT can be obtained because neuronal enzymes involved in 5-HT synthesis (e.g., l-tryptophan hydroxylase) are not fully saturated. Consequently, altering TRP plasma levels increases enzyme saturation and, hence, brain 5-HT synthesis. However, other large amino acids (LNAAs) are transported across the blood-brain barrier by the same transport carrier as TRP. Consequently, brain TRP uptake depends on the TRP/LNAAs ratio (i.e., the relative level of TRP compared to other LNAAs) rather than plasma levels alone (Markus, 2008).

In line with the importance of 5-HT in stress regulation, it has been demonstrated that sub-chronic TRP administration (for seven days) attenuates the larger cortisol stress response observed in the S-allele, thereby diminishing the difference between S- and L-allele carriers (Cerit et al., 2013; Capello and Markus, 2014). It is likely that this results from the importance of 5-HT in regulating negative feedback systems of the HPA axis, since research indicates that sub-chronic TRP administration augments this inhibitory influence (van Praag et al., 2004). Interestingly, effects of TRP and 5-HT functioning seem to be influenced by cognitive stress vulnerability, as TRP augmentation is found to reduce cortisol stress response in stress-prone individuals (Markus et al., 2000). In line with these findings, it might be speculated that TRP also attenuates the difference in sleep quality between S- and L-allele carriers, as previously observed by Brummett et al. (2007), and, hence, that this depends on chronic stress vulnerability.

The present study aims to investigate if the 5-HTTLPR genotype moderates sleep quality as a function of chronic stress vulnerability as measured by neuroticism and, hence, whether this may be influenced by sub-chronic TRP augmentation. In an experimental between-subject design, homozygous S-allele (n = 57) and L-allele carriers (n = 54) with either high or low scores on trait neuroticism were assessed for sleep quality after a seven-day treatment period with either TRP or placebo. Before the start of the treatment, differences in past sleep quality (over the last month) were explored between subject groups. It was hypothesized that: (1) there is a negative association between neuroticism and general past sleep quality, especially in participants with the S’/S’ 5-HTTLPR genotype, and (2) TRP is especially beneficial in reducing the negative effects of stress (neuroticism) on sleep in S’/S’ allele carriers.

## Method

### Participants

Participants were selected from a previously-obtained database of Maastricht University students (n = 771) who were genotyped for 5-HTTLPR using a buccal sample extraction (26% S/S, 47% S/L, 27% L/L) and had already completed an electronic online questionnaire package. This included questions on general health (i.e., smoking and drinking habits, past and present use of medication and psychoactive drugs, and personal or family history of medical or psychiatric complaints) and experience of stressful life events, as well as standardized questionnaires including the Beck Depression Inventory (BDI; see Method section) and the inadequacy (neuroticism) scale of the Dutch Personality Questionnaire (see Measures section below). Subjects were excluded in cases of chronic and/or current illness, current treatment by a psychiatrist, use of psychoactive medication or drugs, excessive use of alcohol (>15 per week), or pregnancy and breastfeeding. In addition, only homozygous S- and L-allele carriers were selected, since differences in stress responses related to 5-HT vulnerabilities are often more pronounced when comparing homozygous genotypes (Way and Taylor, 2010).

Individuals that were able to participate (n = 118) were included in the present study and subsequently divided into high and low trait neuroticism using median split (*Mdn* = 10). This resulted in four groups, including 57 S’/S’ carriers (29 high neuroticism and 28 low neuroticism) and 54 L’/L’ carriers (23 high neuroticism and 31 low neuroticism). Before onset of the study, these participants were first asked to fill out a Pittsburg Sleep Quality Index (PSQI) to measure possible differences in past general sleep quality (past month). During the study, a few participants (n = 6) reported shift work and were therefore excluded for statistical analyses. In addition, participants who missed two or more doses of supplementation (n = 1) were also excluded. The final sample (n = 111) included 19 (17%) men and 92 (83%) women, aged between 22 and 31 years (*M* = 23.9±1.7). The present study was conducted according to the guidelines laid down in the Declaration of Helsinki of 1975, as revised in 1983. The procedures were approved by the Medical Ethics Committee of the Academic Hospital Maastricht (CTCM azM). Informed consent was obtained from all subjects, and participants received payment for their participation in the experiment.

### Design and Procedure

**Figure 1.**
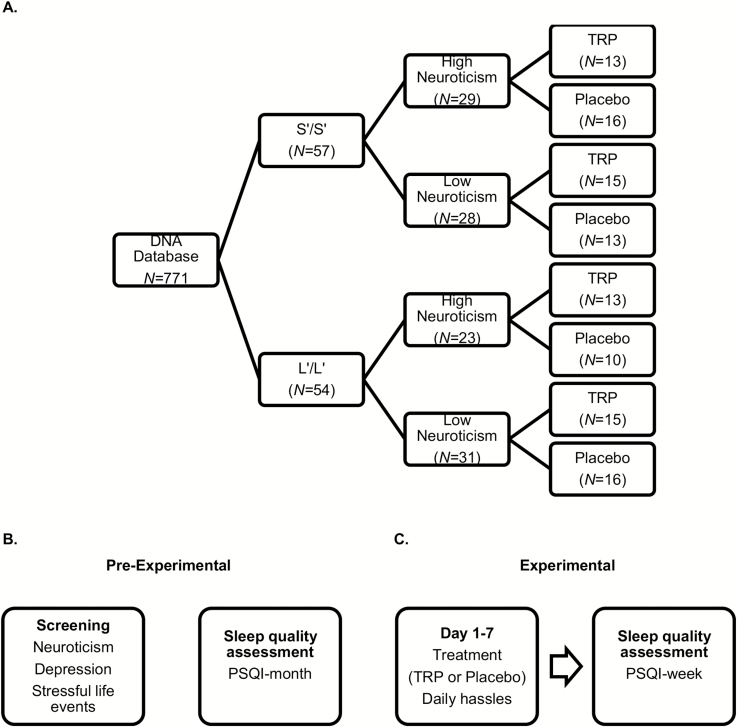
- Experimental design (A), pre-experimental measures (B), and experimental period (C). PSQI: Pittsburg Sleep Quality Index; TRP: tryptophan.

The influence of a seven-day TRP treatment on sleep quality was monitored in participants with S’/S’ and L’/L’ 5-HTTLPR genotypes using a double-blind placebo-controlled design. Participants with S’/S’ and L’/L’ 5-HTTLPR genotypes, further classified as high or low neuroticism, were randomly allocated to either a TRP or placebo condition. After dividing individuals along these conditions, participants were asked to schedule a week in which they were able to participate. A week before testing, all participants picked up an envelope at the university that included instructions, relevant questionnaires, and seven packages of capsules containing daily supplementation. During the test week, participants self-administered capsules containing either TRP or placebo. In order to ensure treatment compliance, participants were instructed to provide a saliva sample every morning, and were led to believe compliance could be assessed by this means. In addition, a questionnaire measuring daily hassles was completed on a daily basis. At day eight, participants visited the university to hand in all materials and complete a PSQI questionnaire to measure their sleep quality during the test week. Figure 1 displays a schematic illustration of the experimental design and procedure.

### Treatment

During the test week, participants were instructed to administer two capsules at a time, three times a day: once each during the morning, afternoon, and evening. Capsules in the two conditions were identical in appearance: however, they contained either tryptophan (0.5g each; for a total of 3.0g/day) or placebo (micorcristaline cellulose; capsules were provided by Elvitaal, Lunteren; The Netherlands). In order to support treatment compliance, a daily checklist was included and participants were instructed to write down the exact time of administration of every pair of capsules. In addition, participants provided a saliva sample every morning, to be stored in the fridge, to be delivered at the end of the experiment, and were led to believe compliance could be assessed using this sample.

### Measures

#### Subjective sleep quality

A slightly modified version of the PSQI (Buysse et al., 1989) was used to assess subjective sleep quality during the treatment week (PSQI-week). Participants had to respond to a variety of statements on a scale ranging from 0 (not during the past week) to 3 (three or more times during the past week). Scoring of the 19 items resulted in 7 component scores, reflecting duration of sleep, sleep disturbance, sleep latency, day dysfunction due to sleepiness, sleep efficiency, overall sleep quality, and medication needed to sleep. The total score of all sub-scores was used as an index of experienced sleep quality during the treatment week, where higher scores are indicative of poorer sleep quality. Before the start of the study, the original version of the PSQI (PSQI-month) was used to explore possible differences in general past-month sleep quality. The PSQI is a well-validated scale with good test-retest reliability (Buysse et al., 1989).

#### Depression

Depressive symptoms were assessed using the BDI, a self-report questionnaire consisting of 21 items that aims to measure presence and severity of depression-related symptoms (Beck et al., 1961). The BDI has been found to have good psychometric properties regarding reliability and validity (Lasa et al., 2000).

#### Stressful Life Events

Lifetime history of stressful life events was assessed using the Dutch Life Events Questionnaire (Kraaij et al., 2003). This scale includes questions about a variety of stressful life events (e.g., divorce of parents, bereavement, victim of a crime). To include more possible life events, the Life Events Inventory (Cochrane and Robertson, 1973) was used as a complementary questionnaire. This scale comprises nine items representing different stressful experiences (e.g., social isolation, change of residence). Scoring of the items resulted in a number of experienced stressful life events (SLE), which was used as an outcome measure.

#### Daily Hassles

In order to assesses changes in daily hassle experiences during the test week, the present study included the Daily Hassles Checklist, adapted from the Illness Management and Recovery Program (Mueser et al., 2006). This questionnaire comprises 20 items and aims to provide an accurate measure of daily hassle experiences.

#### Neuroticism

Neuroticism was assessed using the Inadequacy Scale of the Dutch Personality Inventory (Luteijn and Starren, 1975). This scale comprises a shortened, translated version of the California Psychological Inventory (Gough, 1987). The sub-scale consists of 21 items containing statements intended to measure neuroticism, inadequate feelings, and negativism. Scores range from 0 to 42 and high scores on this scale are closely related to emotional instability and to inefficient ways to cope with negative situations (Magnus et al., 1993; Kendler et al., 2003). Individual outcomes were used to classify participants as having either high or low neuroticism using a median split (*Mdn* = 10).

### Genotyping

In order to determine 5-HTTLPR genotype, sterile swabs (Omni Swabs; Whatman) were used to obtain a buccal cell sample from each participant. Isolation of genomic DNA was performed using QIamp DNA Mini Kits from Qiagen. PCR protocol was followed for the subsequent genotyping (Glatz et al., 2003). Allelic variants were grouped into S’/S’ (S/S, S/L_g_, L_g_/ L_g_) and L’/L’ (L_a_/L_a_). This bi-allelic classification is in line with previous studies (Markus and Firk, 2009; Markus and De Raedt, 2011; Markus and Capello, 2012; Cerit et al., 2013).

### Statistical Analyses

Univariate analyses of variance (ANOVA), using the General Linear Model (SPSS 20.0 for Windows; IBM Corporation), were used to analyze the data regarding the two hypotheses mentioned in the introduction. Analyses regarding treatment-unrelated, general differences in sleep quality included genotype (S’/S’ vs. L’/L’) and neuroticism (high vs. low) as between-subjects factors and PSQI-month scores as the dependent factor. Analyses regarding treatment-related differences during the test week included genotype (S’/S’ vs. L’/L’), neuroticism (high vs. low), and condition (TRP vs. placebo) as between-subjects factors with the PSQI-week score as the dependent variable. Only significant main or interaction effects were interpreted by further post hoc analyses. Sex, age, BDI score, and SLE frequency were initially incorporated as covariates. However, in the final analyses only BDI score was included as a covariate because of its significance. Evaluation of the results was performed using a significance level of 5% (two-tailed). Data are reported as means ± standard deviation.

## Results

### Demographics

As indicated in Table 1, the S’/S’ and L’/L’ 5-HTTLPR group did not differ on a variety of relevant demographic variables, including general sleep quality, depression, neuroticism, and stressful life events.

**Table 1.** Demographics

		**S’/S’**	**L’/L’**	*p*
Men	n	11	8	
Women	n	46	46	
Age	(M ± SD)	23.9±1.5	24±1.8	0.831
Sleep	(M ± SD)	3.7±1.9	3.7±1.6	0.896
Neuroticism	(M ± SD)	11.1±6.3	10.0±6.8	0.374
BDI	(M ± SD)	3.7±3.3	3.4±3.3	0.604
SLE	(M ± SD)	66.9±14.2	67.4±13.9	0.858

Note. BDI: Beck Depression Inventory; M: mean; n: number of participants; SD: standard deviation; SLE: stressful life events.

### Effect of 5-HTTLPR Genotype and Neuroticism on General Sleep Quality

ANOVA with genotype (S’/S’ vs. L’/L’) and neuroticism (high vs. low) as independent variables on the PSQI-month did not reveal significant main or interaction effects. However, a near-significant main effect of neuroticism [*F*(1,103) = 3.708; *p* = 0.057] was observed. Interestingly, it was only when BDI was excluded as a covariate that analyses revealed a significant main effect of neuroticism [*F*(1,104) = 5.948; *p* = 0.016], indicating significantly lower sleep quality (i.e., higher PSQI score) in the high neuroticism group (*M* = 3.18±1.716) compared to the low neuroticism group (*M* = 2.72±1.424) when depressive symptoms are not taken into account.

### Effect of 5-HTTLPR Genotype, Neuroticism, and Tryptophan on Sleep Quality

**Figure 2.**
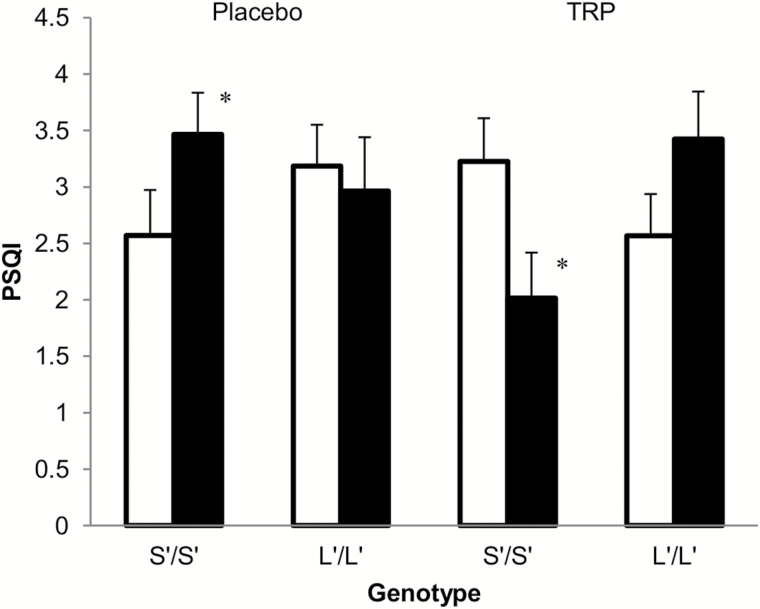
– Estimated marginal means for the Pittsburg Sleep Quality Index (PSQI) score after a week of tryptophan (TRP) or placebo treatment in S’/S’ and L’/L’ genotypes classified as low (white) or high (black) neuroticism. Significant difference between the TRP and placebo conditions: *p < 0.05.

ANOVA with genotype (S’/S’ vs. L’/L’), neuroticism (high vs. low), and condition (TRP vs. placebo) as independent variables on the PSQI-week only revealed a significant genotype x neuroticism x condition interaction [*F*(1,98) = 8.018; *p* = 0.006], indicating a differential effect of treatment on sleep quality in the S’/S’ versus L’/L’ genotypes, depending on neuroticism. Further analyses for both genotype groups only revealed a significant neuroticism x condition interaction in the S’/S’ group [*F*(1,50) = 7.005; *p* = 0.011]. As illustrated in Figure 2, only S’/S’ genotypes with high neuroticism reported significantly better sleep quality (i.e., lower PSQI score) after TRP (*M* = 2.31±1.38) versus placebo (*M =* 3.60±2.26; *p =* 0.021), whereas an opposite effect was reported by S’/S’ carriers with low neuroticism (*p* = 0.045). There were no other main or interaction effects. Moreover, excluding BDI as a covariate did not change the genotype x neuroticism x condition interaction [*F*(1,99) = 7.772; *p* = 0.006].

### Effect of Neuroticism on Stress Experience

In order to check the effectiveness of the neuroticism (high vs. low) classification, an ANOVA was conducted with neuroticism (high vs. low) as the independent variable on SLE frequency. Analyses revealed a significant main effect of neuroticism [*F*(1,109) = 10.399; *p* = 0.002], indicating more past stress experiences in the high neuroticism group (*M* = 71.52±15.69) compared to the low neuroticism group (*M* = 63.29±11.05). In addition, in order to examine the influence of neuroticism on stressful life experiences during the test week, a second ANOVA was performed with neuroticism (high vs. low) as the independent variable on the Daily Hassles Checklist score. This also revealed a main effect of neuroticism [*F*(1,109) = 9.678; *p* = 0.002], indicating the high neuroticism group experienced more stress during the experimental period (*M* = 2.53±1.75) than the low neuroticism group (*M* = 1.56±1.53).

## Discussion

The present study aimed to investigate the interaction between 5-HTTLPR and stress on sleep quality. In contrast to our hypothesis, no differences were found between S’/S’ and L’/L’ carriers on general past sleep quality, regardless of the vulnerability to experience chronic stress (as measured by neuroticism). However, in line with our hypothesis, 7 days of TRP supplementation was exclusively associated with better sleep quality in participants with the S’/S’ 5-HTTLPR genotype who were also classified as high neuroticism.

### 5-HTTLPR Genotype and Stress-Related Sleep Quality

Including trait neuroticism as a valid measure for the vulnerability to frequent, negative stress experiences, it was hypothesized that this would interact with 5-HTTLPR in order to influence sleep quality. To date, only a few studies have examined the influence of 5-HTTLPR on sleep quality, and only one examined whether negative circumstances—as indirectly conceptualized by taking care of a close relative with dementia—may moderate the relationship between 5-HTTLPR and sleep quality (Brummett et al., 2007). Results of this previous study demonstrated that being a caregiver for a spouse or parent with dementia was associated with a reduction in sleep quality, particularly in S-allele 5-HTTLPR carriers. Assuming that this type of caregiving is associated with enhanced physical and emotional strain, the authors concluded that the 5-HTTLPR genotype plays a moderating role in stress-related sleep disturbances. However, although caregiving has been associated with physical and emotional stress, it particularly displays a close relationship with depression (Pinquart and Sörensen, 2003). Brummett et al., (2007) neither controlled for stress nor for depression, it remains unclear from their study whether the reduced sleep quality observed in their S-allele carriers was indeed primarily caused by stress or, instead, by its depressogenic consequences. Depressive symptoms particularly occur as a consequence of long-term uncontrollable/unchangeable negative circumstances and have already been found to be the most important predictor for sleep quality in caregivers for relatives with dementia (Peng and Chang, 2013).

In order to determine whether stress is indeed a moderating mechanism involved, the present study included trait neuroticism as a measure for the vulnerability to frequent stress experiences while controlling for depression (i.e., excluding clinically-relevant BDI scores and including BDI score as a covariate). Results did not reveal the expected interaction between 5-HTTLPR genotype and neuroticism on general past sleep quality, even though high scores on neuroticism were indeed associated with increased frequencies of stressful life experiences and daily hassles. Based on these findings, it seems likely that stress experience itself is not a sufficient factor to influence sleep quality as a function of 5-HTTLPR. Combining the previous findings of Brummett et al. (2007) with our current findings, a more elaborated picture may be reached in which allelic variation in the 5-HTTLPR may influence the effects of stress on sleep quality more precisely by promoting the depressogenic consequence of stress experiences. Of course, this hypothesis can be further supported by including subjects with more profound (i.e., more clinically relevant) depression symptoms. Hence, this might also have been the confounding, uncontrolled factor mediating the interaction between 5-HTTLPR and caregiving on sleep quality as reported by Brummett et al. (2007). Since sleep and depression are closely related (Riemann et al., 2001) and 5-HTTLPR S-allele carriers are already at increased risk to develop depressive symptoms in response to frequent stress experiences (Caspi et al., 2003; Karg et al., 2011), it is suggested that 5-HTTLPR may promote sleep disturbances—particularly in S-allele carriers—when stress is experienced, by way of increasing susceptibility to stress-related depressive symptoms.

### Differential Effect of TRP in 5-HTTLPR Genotype Depending on Neuroticism

In order to examine whether 5-HT vulnerability (as a consequence of the S’/S’ 5-HTTLPR genotype) plays an important role in stress proneness and stress-related sleep quality, the present study additionally examined the effects of brain 5-HT manipulation—by way of TRP augmentation—on sleep quality in relation to the 5-HTTLPR genotype and neuroticism. As expected, sub-chronic TRP supplementation was exclusively associated with better sleep quality in subjects with the S’/S’ 5-HTTLPR genotype who were classified as high neuroticism. Based on previous findings, these beneficial effects of TRP may be related to improved stress adaptation and, hence, reduced stress experience (even though this was not directly measured). After all, S-allele carriers are found to be more prone to experience negative stress, as evidenced in a broad range of epidemiological studies (Risch et al., 2009; Karg et al., 2011) and in acute stress exposure studies (Gotlib et al., 2008; Alexander et al., 2009; Mueller et al., 2010; Way and Taylor, 2010; Markus and De Raedt, 2011), probably as a consequence of 5-HT–receptor sensitization to compensate for lower 5-HTT expression (David et al., 2005; Jans et al., 2007; Markus, 2008). Particularly in combination with chronic stress, 5-HT dysfunction in S-allele genotypes is expected to become worse, causing the stress system to become unbalanced (van Praag, 2004; Markus, 2008 2013). Since TRP is found to increase brain 5-HT synthesis and to reduce stress responsiveness in 5-HTTLPR S-allele carriers (Cerit et al., 2013; Capello and Markus, 2014), its current beneficial effect on sleep quality in S’/S’ genotype subjects with high neuroticism might thus be explained as a result of improved stress adaptation. Nevertheless, since 5-HT is commonly known to play a most important role in stress-related depression (Jans et al., 2007), and 5-HTTLPR is found to moderate the relationship between stress and depression (Caspi et al., 2003; Karg et al., 2011), the current beneficial effects of TRP on sleep quality in S’/S’ carriers might be particularly attributed to its reducing influence on stress-related depressive symptomatology. Tryptophan supplementation is known to increase brain 5-HT synthesis and may therefore compensate for reduced 5-HT functioning and, hence, reduce depressed mood in S’/S’ 5-HTTLPR subjects classified as high neuroticism. This is in line with previous findings demonstrating that TRP augmentation significantly improves mood, especially in 5-HT–vulnerable subjects (Markus and Firk, 2009) and stress-prone individuals (Markus et al., 2000). Furthermore, given the well-established relationship between sleep and depression (Riemann et al., 2001), the ability of TRP to reduce depressed moods is likely to result in a significant improvement of sleep quality. This is in agreement with the previously-described suggestion that the sleep quality reductions in caregivers with the S’/S’ 5-HTTLPR genotype observed by Brummett et al. (2007) are caused by depressive symptomatology, possibly resulting from a diminished functional efficiency of the 5-HT system related to chronic stress experience.

### Limitations

The present study is subjected to the following limitations. First, the small sample size might have reduced statistical power during further analyses of the observed interaction between 5-HTTLPR genotype, neuroticism, and treatment condition. Nonetheless, as expected, separate analyses still revealed a beneficial effect of TRP in participants with the S’/S’ 5-HTTLPR genotype who were classified as high neuroticism. Second, although it was a major objective to isolate the effects of stress from depressive-affective symptoms, exclusion of participants with high BDI scores might have underestimated the effect of neuroticism on sleep quality. It might be possible that, if depression was not controlled for, an interaction between neuroticism and 5-HTTLPR genotype would have been observed. Future studies are needed to disentangle how stress, its depressogenic consequences, and the 5-HTTLPR genotype may interact with each other to exert an influence on sleep quality. In addition, polysomnograpic measures need to be included as additional indices for changes in sleep quality and/or architecture.

### Conclusion

The present study implies that the 5-HTTLPR genotype does not directly moderate the relationship between stress vulnerability and sleep quality. However, allelic variation in the 5-HTTLPR does seem to play an important role in stress-related sleep quality, probably by its moderating influence on the relationship between stress and depressive symptomatology. More specifically, in line with previous findings, it is suggested that in response to chronic stress, individuals with the S’/S’ 5-HTTLPR genotype especially experience depressive symptoms and, hence, related sleep disturbances. These mood alterations are suggested to reflect a decreased functional efficiency of the 5-HT system, resulting from a genetic 5-HT vulnerability further augmented by the negative influence of stress on 5-HT functioning. TRP augmentation may compensate for this reduced 5-HT functioning by increasing brain 5-HT synthesis, thereby reducing depressive symptomatology and, hence, improving related sleep quality. More research is necessary to determine if the potential role of the 5-HTTLPR genotype in regulating sleep quality is indeed caused by its moderating influence on the relationship between stress and depression. In addition, future studies should aim to determine the neurobiological mechanisms responsible for the moderating role of the 5-HTTLPR genotype in the relationship between stress and depression.

## Statement of Interest

None.
